# Systemic immunotherapy of superficial mouse bladder cancer with Avelumab (MSB0010718C), an anti-PD-L1 immune checkpoint inhibitor

**DOI:** 10.1186/2051-1426-3-S2-P70

**Published:** 2015-11-04

**Authors:** Amanda Vandeveer, Jonathan Fallon, Robert Tighe, Helen Sabzevari, Jeffrey Schlom, John Greiner

**Affiliations:** 1 Laboratory of Tumor Immunology and Biology, Center for Cancer Research, National Cancer Institute, National Institutes of Health, Bethesda, MD, USA; 2 EMD Serono Research and Development Institute, Billerica, MA, USA

## 

Bladder cancer is considered a malignancy that is responsive to immunotherapy because of the presence of a (a) high number of somatic mutations, (b) large number of tumor-infiltrating lymphocytes and (c) clinical response to the immune stimulant, Bacillus Calmette-Guerin (BCG). Recent findings of the roles that inhibitory immune receptors and their ligands play in tumor evasion provide some possible explanation as to the limitations of BCG and may offer new therapeutic approaches to some patients with bladder cancer. In this study, an aggressive, bioluminescent orthotopic bladder cancer model, MB49 tumor cells transfected with luciferase (MB49^luc^), was used to study the antitumor effects of an anti-PD-L1 antibody. MB49^luc^ murine tumor cells form multifocal, superficial tumors on the mucosal wall of the bladder reminiscent of superficial human transitional cell bladder carcinomas. MB49^luc^ bladder tumors were shown to constitutively express high PD-L1 levels and the administration of Avelumab, an anti-PD-L1 antibody, induced significant tumor reduction. Antitumor effects subsequently improved overall survival, and both were abrogated by selective *in vivo* depletion of CD4 or CD8 T cells, but not with NK cell depletion. These findings suggest that in this murine bladder tumor model, interruption of the immune suppressive PD-1/PD-L1 complex releases a local adaptive immune response that, in turn, significantly reduces tumor growth. This experimental bladder tumor model can be used to (a) identify host immune mechanisms responsible for tumor resistance or destruction and (b) evaluate combinations of immune-based therapy for bladder cancer which could provide rationale for future clinical studies.

